# From depletion to distress: identifying the nonlinear relationship and core intervention target through computational simulation modelling between compassion fatigue and moral distress among ICU nurses: a cross-sectional study

**DOI:** 10.1186/s12912-025-04196-1

**Published:** 2025-12-04

**Authors:** Xutong Zheng, Xiaoquan Zhu, Aiping Wang

**Affiliations:** 1 Department of Public Service, The First Affiliated Hospital of China Medical University, No.155, Nanjing North Street, Heping District, Shenyang, Liaoning Province China; 2 Department of Intensive Care Unit, The Affiliated Huaian First People’s Hospital of Nanjing Medical University, Huian’an, China

**Keywords:** Compassion fatigue, Moral distress, ICU nurses, Conservation of resources theory, Network analysis, Dose-Response relationship, Bridge symptoms, In silico intervention, Occupational health

## Abstract

**Background:**

Intensive Care Unit (ICU) nurses are at high risk for compassion fatigue and moral distress. Grounded in the Conservation of Resources Theory, this study proposes that compassion fatigue depletes emotional resources, thereby intensifying moral distress. However, the specific pathway and key components driving this relationship remain unclear.

**Objective:**

To investigate the nonlinear relationship between compassion fatigue and moral distress among ICU nurses and to identify core symptoms of compassion fatigue that most significantly influence moral distress.

**Methods:**

A nationwide cross-sectional online survey was conducted from July to December 2023, recruiting 645 ICU nurses from China. Compassion fatigue and moral distress were assessed using the Professional Quality of Life Scale and the Moral Distress Scale-Revised, respectively. The dose-response relationship was analyzed using restricted cubic splines, with moral distress as the outcome and compassion fatigue as the predictor, and network analysis with in silico interventions was applied to identify key compassion fatigue symptoms affecting moral distress.

**Results:**

A significant nonlinear relationship was identified of compassion fatigue on moral distress (nonlinear F = 10.02, *p* < 0.01; adjusted model R² = 0.161), with moral distress increasing sharply at moderate compassion fatigue levels before plateauing. Network analysis and in silico simulations revealed three core compassion fatigue symptoms with the strongest influence on moral distress: “feeling trapped in the helping system” (a burnout symptom), “difficulty remembering important work aspects” (a secondary traumatic stress symptom), and “being a very sensitive person” (a secondary traumatic stress-related trait). Adjusting these symptoms led to significant changes in moral distress levels.

**Conclusion:**

This study confirms a nonlinear relationship of compassion fatigue on moral distress and identifies actionable targets for intervention. Addressing specific compassion fatigue symptoms can disrupt the resource loss spiral, offering a strategic approach to enhancing ICU nurse well-being and care quality.

**Clinical trial number:**

Not applicable.

**Supplementary Information:**

The online version contains supplementary material available at 10.1186/s12912-025-04196-1.

## Introduction

Intensive Care Unit (ICU) nurses are the linchpins of critical care, operating in environments characterized by high acuity, technological complexity, and profound ethical challenges [1, 2]. They are relentlessly exposed to human suffering, death, and situations that often pit their professional ethics against institutional constraints or futile care demands [3, 4]. This perpetual high-stakes environment renders them exceptionally vulnerable to two pervasive and interlinked occupational hazards: compassion fatigue and moral distress [5–7].

Compassion fatigue, a form of profound emotional and physical exhaustion arising from the cumulative strain of empathetically engaging with traumatized patients, signifies a critical depletion of a caregiver’s inner resources [8, 9]. Concurrently, moral distress—a phenomenon first described by Andrew Jameton—occurs when nurses consciously identify the ethically appropriate action to take but are prevented from executing it due to external barriers, such as institutional policies, hierarchical structures, or perceived powerlessness [10, 11]. The resultant psychological disequilibrium is not merely a personal burden; it is a significant professional issue implicated in burnout, reduced quality of patient care, high staff turnover, and the current global nursing shortage [12–14].

A growing body of evidence suggests a compelling correlation between compassion fatigue and moral distress [7, 15, 16]. However, the predominant reliance on conventional correlation analyses in existing literature presents a significant limitation. These studies often treat both constructs as monolithic, aggregate scores, thereby obscuring the underlying mechanistic pathways through which one exacerbates the other. This macro-level approach fails to answer critical questions: Is the relationship linear, or does it exhibit a threshold or saturation effect? More importantly, which specific facets of compassion fatigue are the most potent drivers of moral distress? Understanding these nuances is not an academic exercise; it is a prerequisite for designing precise, effective, and scalable interventions to disrupt this debilitating cycle.

The Conservation of Resources (COR) Theory offers a powerful theoretical lens to illuminate this pathway [17–19]. COR Theory posits that individuals strive to obtain, retain, foster, and protect valued resources, which can be material, conditional, personal, or energy-related. In the context of ICU nursing, key resources include emotional energy, psychological resilience, and a sense of professional efficacy. Psychological stress and maladaptive outcomes occur when these resources are threatened with loss, are actually lost, or when investment of resources fails to yield adequate gain. We conceptualize compassion fatigue as representing a substantial net loss of these emotional and psychological resources, arising from the cumulative strain of empathetically engaging with traumatized patients. This state of resource depletion, in turn, cripples a nurse’s cognitive and emotional capacity to effectively navigate complex ethical dilemmas, making them more vulnerable to the perceived institutional, hierarchical, or futility-related constraints that trigger moral distress [10, 11]. This process initiates a self-perpetuating “loss spiral” [18, 19], wherein the initial resource loss (compassion fatigue) begets further resource loss (intensified moral distress), leading to increasingly negative psychological and professional outcomes. Despite its explanatory power, the application of COR Theory to empirically model this specific resource loss spiral between compassion fatigue and moral distress remains underexplored.

From this theoretical grounding, we derive two key hypotheses. First, we hypothesize that the relationship between compassion fatigue and moral distress is nonlinear. Specifically, we predict a saturation effect: moral distress will increase sharply at moderate levels of compassion fatigue as resources are depleted, but the rate of increase will plateau at high levels of compassion fatigue, as the nurse’s emotional responsiveness diminishes due to near-total resource exhaustion. To test this hypothesis, we employ restricted cubic spline (RCS) regression, which is uniquely suited to model and visualize such complex, nonlinear dose-response relationships without assuming linearity a priori [20, 21].

Second, COR Theory suggests that not all resource losses are equal. Certain core aspects of compassion fatigue may be more consequential in propelling the loss spiral toward moral distress. Therefore, our second hypothesis is that specific, identifiable symptoms within the compassion fatigue network act as central “bridge” nodes that exert a disproportionately strong influence on the moral distress network. To deconstruct these complex constructs and identify these critical intervention targets, we utilize network analysis and in silico intervention techniques [22, 23]. This approach allows us to move beyond aggregate scores and pinpoint the precise symptoms whose modification would most effectively disrupt the network and reduce moral distress.

The context of critical care nursing in China, where this study was conducted, adds a layer of urgency to this investigation. Chinese ICUs are often characterized by high patient-to-nurse ratios and a hierarchical medical culture [24–26]. These factors can contribute significantly to the workload and psychological demands on nurses, potentially intensifying the resource depletion central to compassion fatigue, while also creating situational constraints that can trigger moral distress. Investigating the CF-MD pathway within this context is therefore highly relevant.

To address the aforementioned gaps, this study employs a novel, multi-method analytical approach grounded in COR Theory. We move beyond simple correlation to: (1) model the dose-response relationship between compassion fatigue and moral distress using restricted cubic spline (RCS) regression, testing for non-linearity across the full spectrum of experiences; and (2) utilize network analysis and in silico intervention techniques to deconstruct these constructs into their constituent symptoms, identifying the most central and influential “bridge” symptoms within the compassion fatigue network that actively propel the moral distress network.

The primary aim of this study is to elucidate the mechanistic pathway through which compassion fatigue aggravates moral distress among ICU nurses in China. By integrating COR Theory with advanced statistical modeling, we seek to: (1) test for a hypothesized nonlinear, saturating relationship between compassion fatigue and moral distress using RCS; and (2) identify the core, actionable symptoms of compassion fatigue that most actively drive moral distress through network analysis and in silico simulations. Our findings are expected to provide a robust evidence base for developing targeted strategies to mitigate this destructive cycle, thereby safeguarding nurse well-being and, ultimately, ensuring the sustainability and quality of critical care.

## Methods

### Study design

This is a cross-sectional survey conducted and reported in accordance with the Strengthening the Reporting of Observational Studies in Epidemiology (STROBE) statement.

### Study setting and sampling

This study used a hybrid multi-center convenience and snowball sampling strategy to recruit targeted 578 ICU nurses from 17 provinces in China, focusing on tertiary Grade A hospital ICUs. While this approach was pragmatically chosen to facilitate nationwide recruitment where probabilistic sampling was not feasible, we acknowledge it may introduce potential selection biases, such as homophily (where nurses with shared experiences or higher distress levels might be more likely to participate) and site clustering (where responses from a few active recruitment sites could be over-represented).

To mitigate these potential biases, we implemented several strategies: (1) we broadened the recruitment network across diverse geographical and institutional settings to maximize sample heterogeneity; (2) we applied strict, uniform inclusion and exclusion criteria to ensure a functionally homogeneous population of eligible frontline ICU nurses; and (3) in our statistical models, we adjusted for key demographic and occupational covariates that could be confounded with the sampling method. Data were collected from July to December 2023 via an online platform (Wenjuanxing). Trained data collectors ensured quality, and participants provided informed consent. An a priori sample size calculation was performed to ensure adequate power for the primary analyses. For the restricted cubic spline (RCS) regression, a power analysis was conducted using G*Power 3.1 for a multiple linear regression model (effect size f² = 0.10, α = 0.05, power = 0.95, 10 predictors), which indicated a minimum sample size of 254. For the network analysis, a widely recognized heuristic of 10 participants per network node was applied. Given our network comprised 52 nodes (from the ProQOL and MDS-R scales), a minimum of 520 participants was required. Accounting for approximately 10% potential invalid responses, the target sample size was set to 578. For more detailed information related to the implementation framework, please refer to the supplementary file 1.

### Inclusion and exclusion criteria

**Inclusion Criteria**: (1) Registered nurse qualification: Nurses holding a valid nursing license; (2) Workplace and role: Currently employed in a clinical role providing direct patient care in a general intensive care unit (ICU) or specialized ICU (e.g., cardiac ICU, neurological ICU, respiratory ICU, surgical ICU, etc.); (3) Work experience: At least 3 months of clinical experience in an ICU setting; (4) Informed consent: Provided informed consent to participate.

**Exclusion Criteria**: (1) Non-full-time/non-permanent ICU Roles: Nurses temporarily assigned or rotated to the ICU from non-ICU departments; (2) Long-term leave: Nurses on extended leave (e.g., maternity leave, sick leave) who were not actively working in the ICU during the data collection period; (3) Non-direct clinical roles: Nurses in the ICU performing purely administrative, managerial, teaching, or research duties without direct patient care responsibilities, even if their title remains “nurse.”(4) Inability to complete the survey: Individuals with language barriers, cognitive impairments, or other severe conditions that prevent them from understanding the questionnaire content or independently completing the online survey.

### Ethics

The study protocol, including sampling methods, participant recruitment, electronic informed consent procedures, and data security measures, was evaluated and approved by the Ethics review board of Huai’an first people’s hospital affiliated to Nanjing Medical University (Approval No.: KY-2023-087-01). The research was conducted in strict compliance with the Declaration of Helsinki.

### Instrument

#### Demographic information

Information regarding participants’ gender, age, marital status, years worked in the ICU, educational background, professional title, monthly income (CNY), and satisfaction with income was collected.

#### Compassion fatigue scale

Professional quality of life was assessed using the 30-item professional quality of life scale (ProQOL), a widely used and validated tool for measuring the positive and negative aspects of working in helping professions [27, 28]. The scale consists of three 10-item subscales: Compassion satisfaction, burnout, and secondary traumatic stress (STS).

In line with the conceptualization of compassion fatigue as a construct encompassing the negative effects of caregiving—specifically, work-related exhaustion (burnout) and trauma-related symptoms stemming from exposure to patients’ suffering (STS)—we operationalized a composite compassion fatigue score [29, 30]. This approach aligns with the conservation of resources theory, as it captures the comprehensive depletion of emotional and psychological resources central to our hypothesis.

Each item is rated on a 5-point likert scale (1 = never, 5 = very often). To ensure that a higher total score consistently reflects a higher level of compassion fatigue (i.e., greater resource depletion), we applied the following scoring procedure: the burnout and STS subscales were scored as per the standard manual, where higher scores indicate greater distress. For the compassion satisfaction subscale, items were reverse-scored so that a higher score indicates lower satisfaction, or compassion dissatisfaction. The total compassion fatigue score was then computed as the sum of the burnout, STS, and reversed compassion satisfaction subscale scores, resulting in a potential range of 30 to 150, with higher scores indicating more severe compassion fatigue. This scoring method enhances the unidimensional interpretation of the composite variable for subsequent dose-response and network analyses.

#### Moral Distress Scale-Revised (MDS-R)

Moral distress was measured using the 22-item Moral Distress Scale-Revised (MDS-R), a validated tool assessing morally distressing situations among healthcare professionals [31, 32]. Revised from Corley’s original scale, the MDS-R was updated to enhance clarity and applicability across inpatient settings, including ICUs. Each item is scored on two 0–4 scales for frequency (0 = never, 4 = very frequently) and intensity (0 = none, 4 = great extent). A composite score (0–352) is calculated by multiplying frequency and intensity scores per item and summing across all items.

### Statistical analysis

#### Descriptive statistical analyses

Descriptive statistics were conducted using IBM SPSS 29.0. No missing data were present in the final dataset for the analyzed variables, as the online survey platform required responses to all items. Continuous variables were reported based on their distributional properties: normally distributed variables were expressed as means with standard deviations, whereas non-normally distributed variables were summarized using medians and interquartile ranges. For categorical variables, frequencies and their respective percentages were used to describe the data.

#### Estimation of the dose-response relationship between MD and CF

To explore the dose-response relationship of moral distress (MD) as a function of compassion fatigue (CF) among ICU nurses, we employed restricted cubic spline regression using the **rms** package in R [20, 21]. Two models were constructed: (1) **Model 1**: Assessed the association between MD and CF using RCS with four knots, without adjusting for covariates. (2) **Model 2**: Extended Model 1 by adjusting for potential confounding variables, including gender, age, marital status, working time in ICU, educational background, professional title, monthly income, and satisfaction with income.

For both models, we fitted RCS with four knots placed at the default quantiles of MD. Standardized coefficients were calculated after Z-score normalization of MD and CF to allow for effect size comparison [20, 21, 33]. Model fit was evaluated using the R-squared and adjusted R-squared statistics. The significance of nonlinear associations was examined using ANOVA F-tests.

To visualize the non-linear association between moral distress (MD) and compassion fatigue (CF), we applied restricted cubic spline regression models with four knots. Predicted values of MD were plotted against the range of MD scores with and without adjustment for covariates. The models were built using the **rms** package in R, and the **Predict()** function was used to compute fitted values and 95% confidence intervals (CI). Spline plots were generated using the **plot()** function to graphically represent the dose–response relationship between MD and CF. Curves of model 1 and model 2 were both drawn.

#### Identify the core intervention target between CF and MD

We processed all data using R 4.2.1 in RStudio with the packages “readxl,” “bootnet,” “networktools,” “nodeIdentifyR,” and “dplyr.” No missing value processing was needed as the dataset was complete. Moral distress and compassion fatigue items were conceptualized as nodes, forming two sub-networks: a 22-node moral distress sub-network and a 30-node compassion fatigue sub-network, creating a 52-node “MD-CF network.” The Ising model was fitted to simulate interventions by adjusting the intercept term of logistic regressions for each node. For the moral distress scale, raw scores were calculated by multiplying frequency and distress, with products of zero scored as 0 and others as 1. For the compassion fatigue scale, a 5-point Likert scale, “never experienced” (score 1) was coded as 0, and “rarely” to “always” (scores 2–5) as 1 [22, 23].

We used the bootnet package’s estimateNetwork function to fit the Ising model for the total population, arranging nodes in a “spring” layout, which was reused for other networks to ensure comparability [22, 23]. To identify critical bridge symptoms in the compassion fatigue sub-network affecting moral distress, we calculated four bridge centrality indices: bridge strength (total connectivity to other communities), bridge expected influence (sum of connectivity to other communities), bridge closeness (average distance to nodes in other communities), and bridge betweenness (frequency on shortest paths between communities). Stability of these indices was assessed using the central stability coefficient (CS coefficient), which measures consistency as data subsets increase, with CS > 0.5 indicating strong stability, 0.25–0.5 acceptable, and < 0.25 poor [22, 23].

We then conducted computer-simulated interventions using the nodeIdentifyR algorithm (NIRA), which systematically varies threshold parameters. NIRA generated 5,000 simulated observations per intervention, including one for original thresholds and one per symptom-specific intervention, using the IsingSampler package with the Metropolis-Hastings algorithm for data generation [22, 23]. Interventions were either alleviating (reducing thresholds) or aggravating (increasing thresholds), with effect sizes set at 2 standard deviations based on prior studies. We simulated both intervention types for each compassion fatigue sub-network node, calculated mean moral distress values post-intervention, and plotted anxiety changes across 31 simulations (1 original + 30 interventions) [22, 23]. Differences in compassion fatigue levels between each intervention and the original were tested using multiple independent samples t-tests, corrected for fdr multiple comparisons.

## Results

### Results of descriptive statistical analyses

From Table 1, a total of 887 ICU nurses were approached for this study, of whom 645 submitted completed questionnaires, resulting in a response rate of about 72.7%. The remaining 242 nurses refused to take part in the study. Females constituted the vast majority (88.4%), and most participants were under 40 years old (92.2%). A large proportion held undergraduate qualifications (76.4%). Regarding job titles, nurse practitioners (44.8%) and nurses-in-charge (31.9%) were the most represented. Over half (51.0%) had five or fewer years of ICU experience, while very few (1.9%) had over 21 years. Monthly earnings typically fell between 4001 and 8000 yuan (52.4%), though 56.0% were not satisfied with their compensation. A considerable number (65.0%) indicated an intention to resign, and 2.8% had resigned previously.

**Table 1:** Characteristics of participants (categorical variable)

Gender	*N*	Percentage (%)
male	75	11.6
female	570	88.4
Age		
18–29	300	46.5
30–39	295	45.7
40–49	46	7.1
50–59	4	0.6
Marital status		
married	392	60.8
unmarried	246	38.1
divorced	6	0.9
other	1	0.2
Working time in ICU		
≤ 5 years	329	51.0
6–10 years	178	27.6
11–15 years	104	16.1
16–20 years	22	3.4
≥ 21 years	12	1.9
Education level		
college level	134	20.8
undergraduate	493	76.4
post-graduate	16	2.5
doctorate	2	0.3
Professional title		
nurse	130	20.2
nurse practitioner	289	44.8
Nurse-in-charge	206	31.9
Associate Chief Nurse	17	2.6
Chief Nurse	3	0.5
Monthly income		
≤ 4000	91	14.1
4001–8000	338	52.4
8001–12,000	183	28.4
12,001–16,000	27	4.2
16,001–20,000	5	0.8
≥ 20,001	1	0.2
Satisfaction with income		
satisfied	284	44.0
unsatisfied	361	56.0
Turnover intention		
never	208	32.2
have the intention to leave	419	65.0
resigned once	18	2.8

As in Table 2, assessment of continuous variables showed deviations from normality (all Shapiro-Wilk *p* < 0.001). The median moral distress score was 53.0 (IQR: 65.00), ranging broadly from 0 to 352. Compassion fatigue had a median of 82.0 (IQR: 24.00), with values between 35.0 and 139.

**Table 2:** Characteristics of participants (continuous variable)

Variable name	Median	IQR	Minimum	Maximum	Shapiro-Wilk	
					W	*p*
Moral distress total score	53.0	65.00	0.0	352	0.797	< 0.001
Compassion fatigue total score	82.0	24.00	35.0	139	0.944	< 0.001

### Dose-response relationship between MD and CF

The unadjusted RCS regression model (Model 1) showed a significant nonlinear association of moral distress (MD) with compassion fatigue (CF). The effect of CF on MD across its range was statistically significant, with an estimated total effect of **56.43** (SE = 6.56, 95% CI: 43.57–69.30). The nonlinear component of the spline was also significant (*F* = 10.02, *p* < 0.01), confirming a non-linear relationship. The model explained approximately **13.13%** of the variance in CF (*R*^2^ = 0.131), suggesting a moderate effect size. Standardized regression analysis showed that the first nonlinear term and second nonlinear term had a significant effect (*β* = 0.79, *p < 0.01 and β* = -5.37, *p* < 0.01), although the linear term were not significant individually (*p* = 0.56).

After adjusting for all covariable, Model 2 revealed that the non-linear association of CF on MD remained statistically significant (*F* = 8.06, *p* < 0.01), with a total effect estimate of **53.17** (SE = 6.81, 95% CI: 39.81–66.53). The adjusted model explained a greater proportion of the variance in MD (*R*^2^ = 0.161), and the standardized coefficient for CF’s main spline term was **β = 0.71**, *p* < 0.01. Among covariates, only gender was statistically significant (< 0.001).

**Fig. 1:**
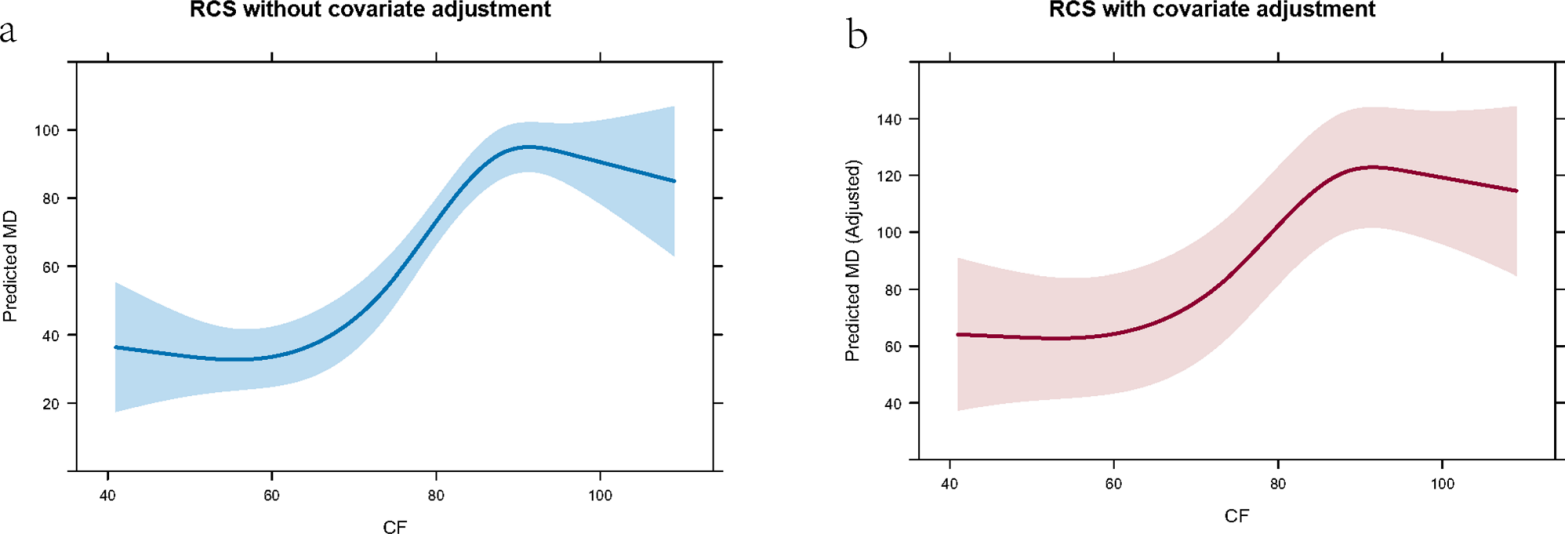
Restricted cubic spline curve for the association between compassion fatigue and moral distress: a without covariate adjustment, b with covariate adjustment

Figure 1 Restricted cubic spline plots showing the association between compassion fatigue and predicted moral distress among ICU nurses. The plot on the left, demonstrating how MD levels change in response to CF, illustrates the unadjusted association, with MD increasing sharply at middle CF levels (about 60–90) and plateauing at higher or lower CF values. This suggests a potential saturation effect where additional increases in compassion fatigue are associated with smaller incremental increases in moral distress in the lower or higher level. The plot on the right presents the covariate-adjusted association. While the adjusted model shows a similar trend, the relationship is slightly attenuated, indicating that some of the association is explained by the covariates. The shaded areas represent the 95% confidence intervals in both plots. The confidence bands widened at the extremes of CF, indicating greater uncertainty due to fewer observations in those ranges.

### Results of in silico interventions

**Fig. 2:**
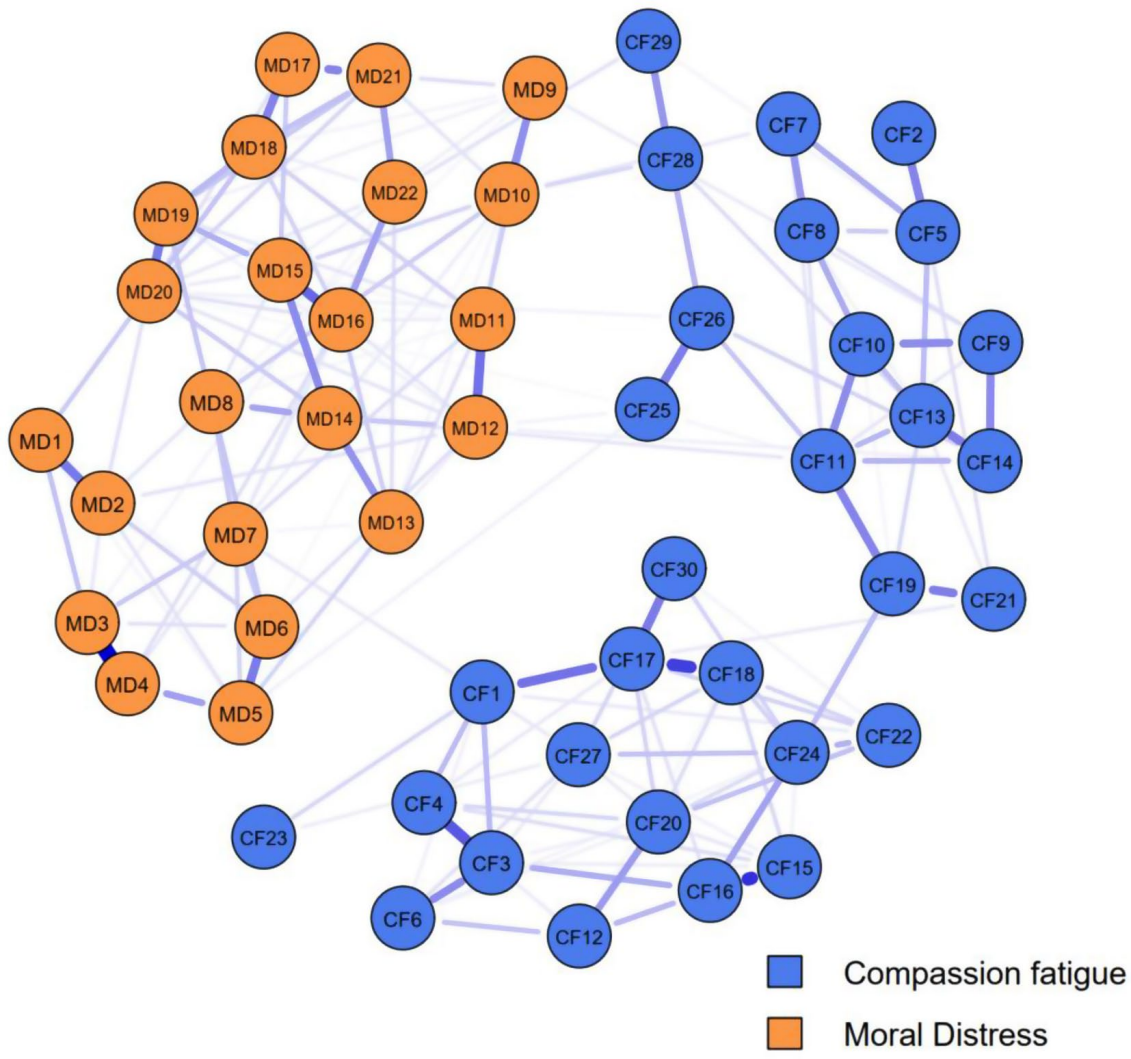
Network plot

**Fig. 3:**
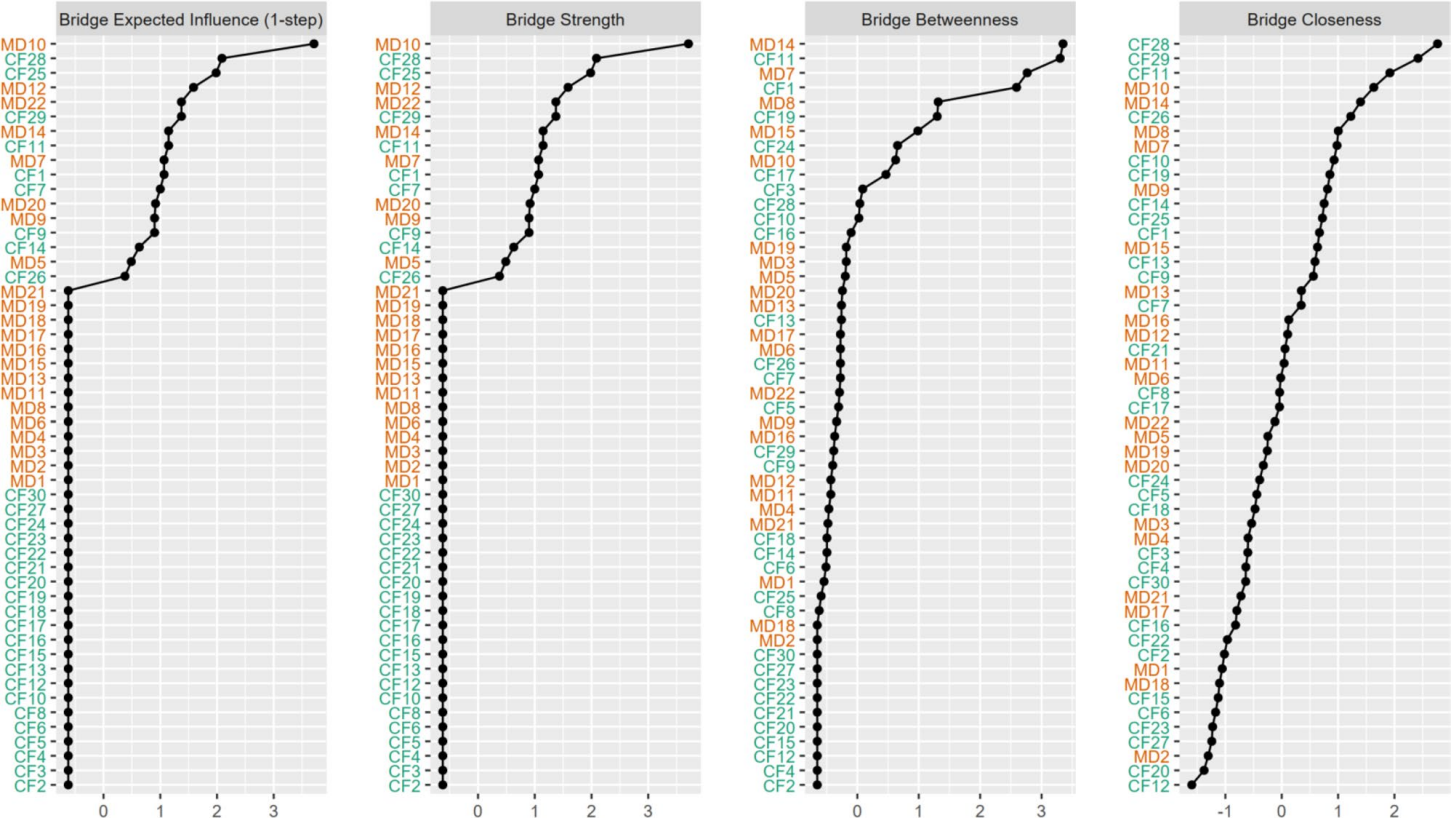
Indices of network analysis

**Fig. 4:**
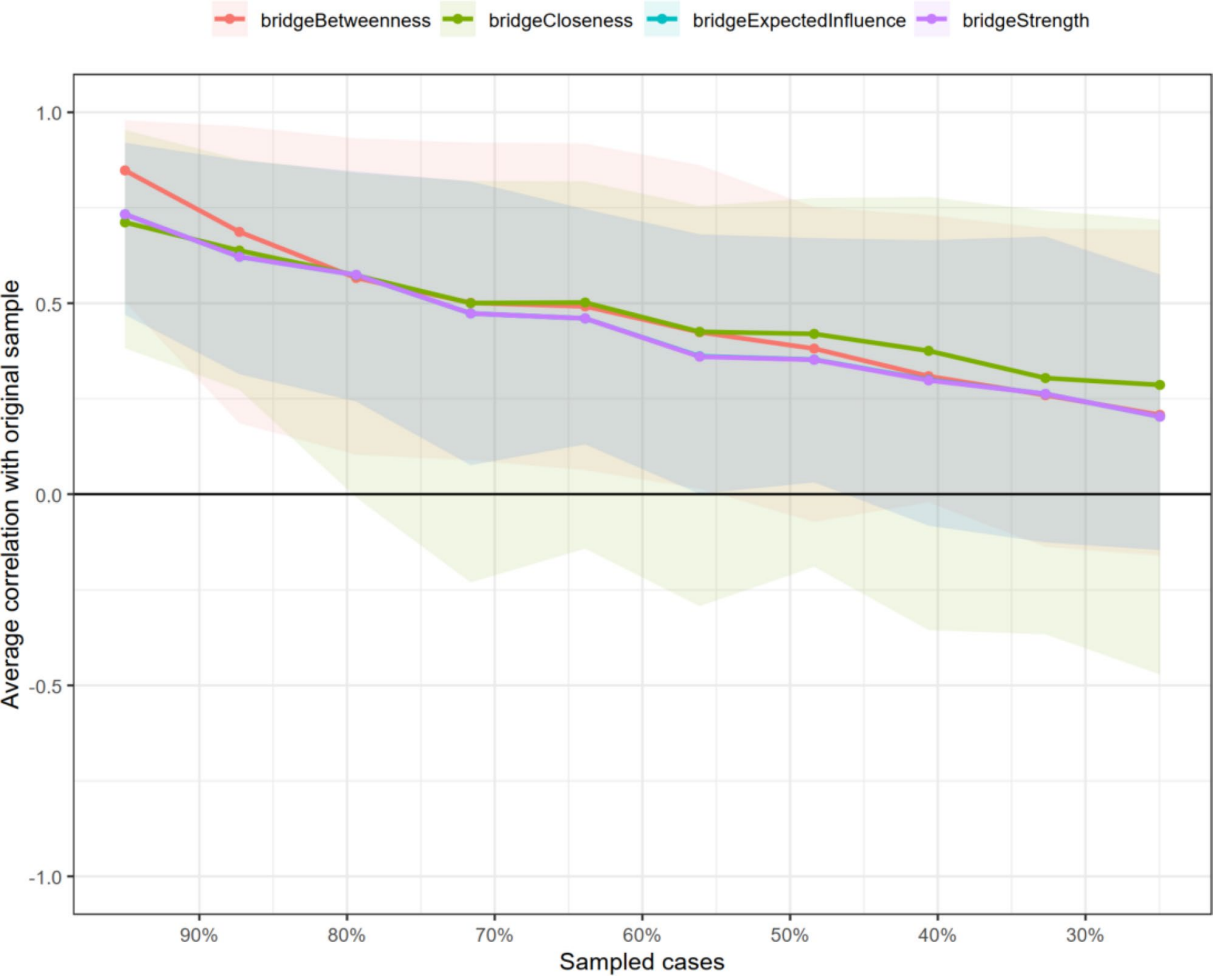
Stability assessment of indices

The network analysis of MD-CF items revealed a connected structure among the 52 items (Fig. 2). The network structure reveals several key connections. For instance, strong positive relationships (indicated by thick blue lines) are visually apparent between nodes such as MD1 and MD2, as well as between CF17 and CF18. Centrality indices (Fig. 3) showed MD10 with the highest expected influence and strength, followed by CF28; closeness and betweenness were highest for CF28 and MD14 respectively, indicating these items’ key roles in activating the network. Stability assessment via case-dropping bootstrap (Fig. 4) demonstrated relative robust centrality estimates: average correlations with the original sample remained approximately 0.25 when retaining ~ 30% of cases for betweenness and closeness (CS-coefficient >0.25), but slightly lower for bridge strength (CS-coefficient <0.25), suggesting relative poor strength and influence measures.

**Fig. 5:**
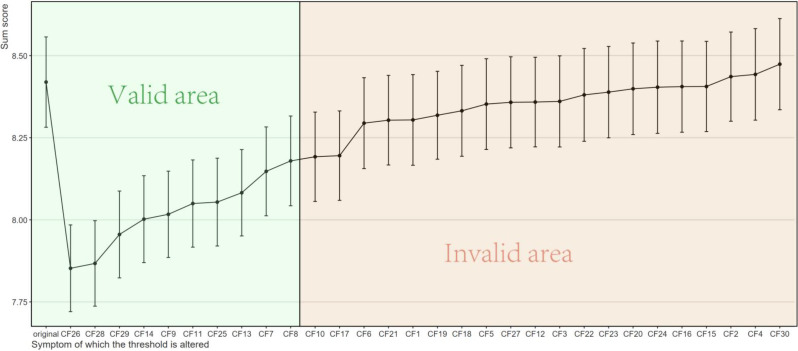
Simulated alleviating interventions results

**Fig. 6:**
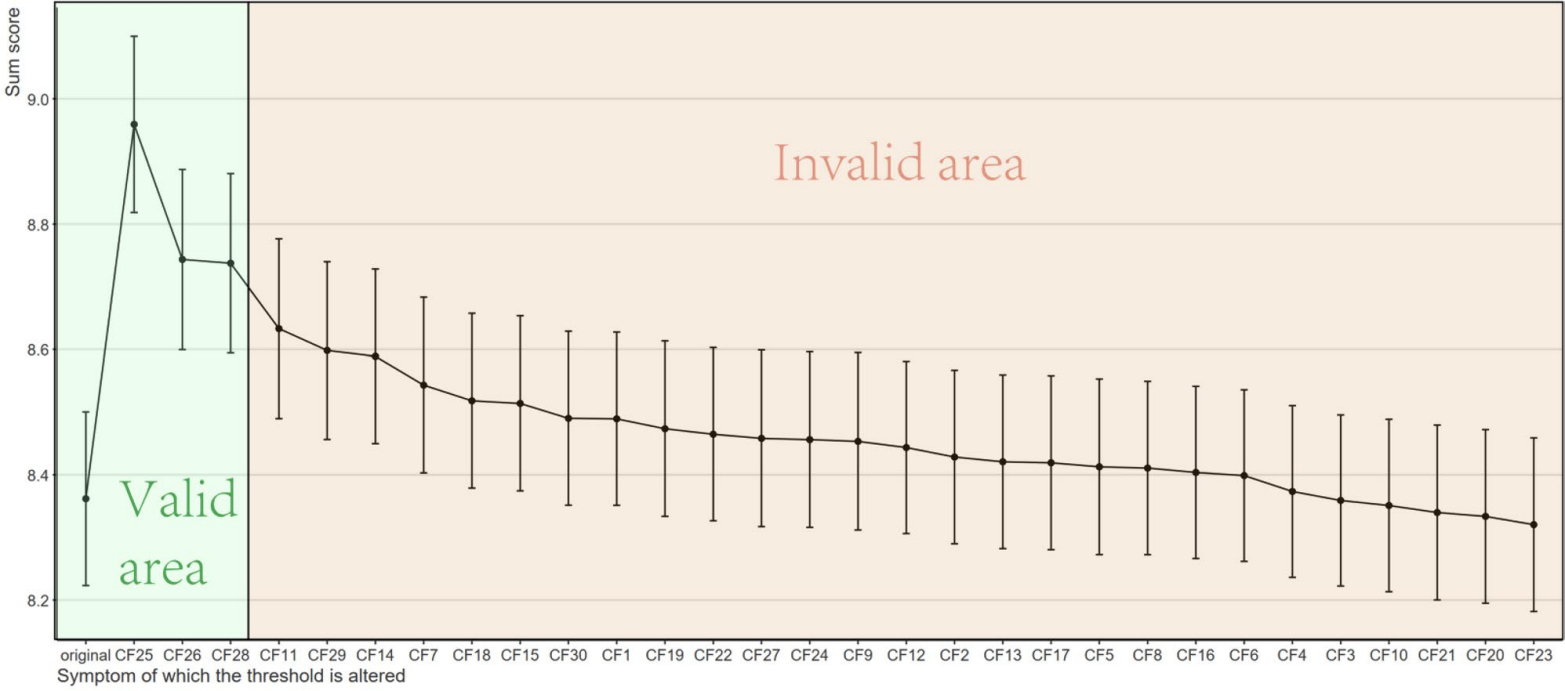
Simulated aggravating interventions results

To evaluate which nodes in the compassion fatigue sub-network of ICU nurses are critical in contributing to moral distress, we conducted computer-simulated interventions (in silico interventions) on all nodes within the compassion fatigue sub-network, comparing changes in the total moral distress score under both alleviating and aggravating intervention scenarios. First, we performed alleviating interventions to simulate the effect of reducing the value of specific compassion fatigue nodes on the total moral distress score. As shown in the figure, reducing the values of 10 compassion fatigue nodes theoretically led to changes in the total moral distress score (indicated by the green area in the Fig. 5), with statistically significant results. The top three nodes were CF26 (“I feel trapped in a system of helping”), CF28 (“I have difficulty remembering important aspects of my work with trauma victims”), and CF29 (“I am a very sensitive person”). Subsequently, we conducted aggravating interventions, which, in contrast to alleviating interventions, aimed to simulate the effect of increasing the value of specific compassion fatigue nodes on the moral distress score. As illustrated in Fig. 6, increasing the values of three compassion fatigue nodes theoretically resulted in changes in the total moral distress score (indicated by the green area in the figure), with statistically significant results. These three nodes were CF25 (“Due to my helping work, I have intrusive, frightening thoughts”), CF26, and CF28.

## Discussion

This study, grounded in the Conservation of Resources Theory, utilized a cross-sectional survey integrated with advanced network analysis to elucidate the mechanistic pathway through which compassion fatigue exacerbates moral distress among ICU nurses. Our principal findings reveal a significant non-linear dose-response relationship between CF and MD and successfully identify specific core symptoms within the CF network that act as potent bridges to the MD network. These insights provide a robust empirical foundation for developing precise interventions aimed at mitigating this debilitating cycle. As predicted by COR Theory, our findings collectively depict a classic ‘resource loss spiral’: the emotional resource depletion characterized by compassion fatigue leaves nurses with insufficient capacity to navigate ethical challenges, thereby intensifying moral distress, which in turn represents a further psychological resource loss.

### Key findings and interpretation

The findings from our macro-level (dose-response) and micro-level (network) analyses are not merely parallel results but are intrinsically complementary, offering a multi-layered understanding of the CF-MD relationship. The nonlinear dose-response curve maps the overall terrain of this relationship, while the network analysis illuminates the specific pathways and mechanisms that traverse this terrain. We propose that the sharp increase in moral distress observed at moderate levels of compassion fatigue (approximately 60–90 points) corresponds to the critical activation of a cluster of highly influential bridge symptoms within the CF network. As compassion fatigue depletes resources to this threshold, symptoms such as feeling ‘trapped in the system’ (CF26) and experiencing ‘cognitive difficulties’ (CF28) may become particularly salient and potent, acting as powerful drivers that rapidly propel the nurse into higher states of moral distress. Conversely, the subsequent plateau at high CF levels may reflect a state of network saturation or systemic shutdown, where the additional impact of these specific bridges diminishes as the entire psychological system becomes overwhelmed. Thus, the network model provides a plausible mechanistic explanation for the shape of the macroscopic dose-response curve.

#### Non-linear Dose-Response relationship: saturation effect and resource depletion

Our primary finding is a significant non-linear association between compassion fatigue and moral distress. Both the unadjusted and covariate-adjusted restricted cubic spline models confirmed that this relationship is not linear. Specifically, moral distress levels increased sharply at moderate levels of compassion fatigue (approximately 60–90 points) but plateaued at both lower and higher extremes of CF.

This non-linear pattern can be effectively interpreted through the lens of COR Theory’s loss spiral and a saturation effect [34–36]. Initially, as compassion fatigue increases (representing a continual loss of emotional resources), nurses’ reservoir of psychological resources is progressively depleted, severely diminishing their capacity to navigate ethical challenges. At this stage, even common ethical dilemmas can trigger intense moral distress, leading to the rapid rise in MD scores—a classic manifestation of a resource loss spiral. However, at very high levels of compassion fatigue, nurses’ emotional resources may be nearly completely exhausted, potentially leading to a state of emotional numbing or psychological shutdown. In this state, their reactivity to subsequent ethical stimuli is diminished, and the rate of increase in moral distress levels plateaus, demonstrating a saturation effect [37–39]. This finding advances beyond previous studies that reported only linear correlations by providing a more precise, dynamic depiction of their relationship. It suggests that the critical window for intervention likely exists in the ascending phase before compassion fatigue reaches its peak severity. This study also enriched the COR theory’s detailed depiction of the relationship between psychological resources and health outcomes.

#### Core Bridge symptoms: targets for precise intervention

The most innovative finding of this study stems from the network analysis and in silico interventions, which revealed that specific components (nodes) of compassion fatigue have a differential impact on moral distress.

Implications of alleviating interventions: Our simulations demonstrated that effectively reducing the severity of specific CF nodes through targeted interventions (e.g., cognitive-behavioral therapy, mindfulness training, enhanced structural support) would theoretically lead to a significant reduction in overall moral distress scores. The top three nodes identified for alleviating interventions were CF26 (“I feel trapped by my helping role”), CF28 (“I have difficulty remembering important aspects of my work with trauma victims”), and CF29 (“I am a very sensitive person”). This indicates that successful interventions focusing on these specific symptoms could yield the highest leveraged effect in interrupting the resource loss spiral and alleviating moral distress. For instance, addressing the “systemic entrapment” reflected in CF26 may require organizational changes such as implementing ethical debriefing protocols and increasing nursing autonomy in decision-making [40, 41]. Conversely, tackling the “cognitive avoidance” of CF28 might require individual-level psychological skill training [42, 43].

Preventive value of aggravating interventions: Conversely, the simulations showed that the worsening of CF25 (“Due to my helping work, I have intrusive, frightening thoughts”), CF26, and CF28 would be most effective in exacerbating moral distress. This result confirms the central role of these symptoms from an opposite perspective. Therefore, in clinical management, these nodes should be treated as critical early warning signs. Proactively identifying nurses with elevated scores on these specific items through routine screening (e.g., using the ProQOL) and providing them with preventative support and resource replenishment could, in theory, prevent their moral distress from progressing to a more severe and less reversible state. This embodies a strategy where “preventing worsening is the best treatment.”

### Implications for clinical practice and policy

The findings of this study have direct implications for ICU nursing management and the promotion of occupational health:

 1.Screening and early warning: Healthcare institutions should implement regular screening for compassion fatigue and moral distress among ICU nurses. Moving beyond aggregate scores, screening should focus on monitoring the identified core bridge symptoms (e.g., CF25, CF26, CF28, CF29). An increase in these specific symptoms should serve as a red flag for heightened psychological risk [44, 45].

 2.Precision interventions: Interventions should be precisely designed to target the identified core bridge symptoms, moving beyond a one-size-fits-all approach.

Targeting CF26 (“I feel trapped in a system of helping”): This symptom reflects a sense of systemic entrapment and powerlessness. Interventions should therefore focus on organizational and leadership reforms [40, 41]. This includes implementing structured ethical debriefing sessions to process morally challenging events, clearly defining and expanding nursing autonomy in clinical and ethical decision-making, and fostering transparent communication channels with management to address systemic constraints.

Targeting CF28 (“I have difficulty remembering important aspects of my work with trauma victims”) and CF25 (“Due to my helping work, I have intrusive, frightening thoughts”): These symptoms are core indicators of secondary traumatic stress, involving cognitive avoidance and intrusive memories. Interventions should aim to directly process traumatic content and build resilience. This can be achieved through trauma-informed care training and access to evidence-based therapies such as Eye Movement Desensitization and Reprocessing (EMDR) or Trauma-Focused Cognitive Behavioral Therapy (TF-CBT) [42, 43, 46].

Targeting CF29 (“I am a very sensitive person”): This trait, while potentially contributing to empathy, requires skilled management to prevent it from becoming a vulnerability. Interventions should focus on helping nurses reframe this sensitivity as a strength that needs protective strategies. This can be facilitated through emotional regulation training and mindfulness-based stress reduction (MBSR) programs, which equip individuals with skills to observe and manage emotional responses without becoming overwhelmed [47, 48].

 3.Organizational and systemic reforms: While individual-level skill building is crucial, our findings, particularly the centrality of symptoms like feeling “trapped in a system” (CF26), underscore that sustainable change requires concurrent organizational reforms. Healthcare institutions must address the foundational work environment factors that deplete resources and provoke moral dilemmas. Key systemic interventions include:

Optimizing staffing and workload: Implementing evidence-based nurse-to-patient ratios in the ICU is critical to reduce excessive workload, a primary driver of both compassion fatigue and moral distress [49, 50].

Cultivating an ethical climate: Establishing structured forums such as regular ethics rounds or debriefings led by ethicists or trained facilitators can provide a safe space for nurses to discuss ethical challenges, receive support, and collaboratively develop solutions, thereby reducing their sense of isolation and powerlessness [51].

Enhancing leadership support: Strengthening supportive leadership and transparent communication from nurse managers and hospital administration is essential. Leaders who actively listen to frontline concerns, advocate for their staff, and involve them in unit-level decisions can significantly buffer the impact of resource loss and foster a culture of trust and psychological safety [52, 53].

Addressing these macro-level factors is indispensable for creating a work environment that conserves, rather than depletes, the psychological resources of ICU nurses, thereby preventing the onset and escalation of the CF-MD cycle.

### Limitations and future research

This study has several limitations. First, the cross-sectional design could not provide a direction for causal reasoning. Future longitudinal studies are needed to verify the predictive power and causal pathways of these bridge symptoms. Second, the sample was exclusively from China, where cultural specificities (e.g., high power distance, collectivism) may influence the manifestation of moral distress and compassion fatigue, potentially limiting the generalizability of the findings. Cross-cultural validation is necessary. Besides, the network structure is contingent on the items of the chosen scales; future research could incorporate qualitative methods to define a more comprehensive set of nodes. Finally, regarding our network analysis, the case-dropping bootstrap indicated that the bridge strength centrality index was unstable. This implies that while we can be confident in the identity of the very highest-centrality nodes identified through in silico interventions, the precise rank order of all nodes by bridge strength is uncertain. Therefore, our interpretations focused on the most influential nodes as identified by the more robust in silico simulation method and the bridge expected influence index (which showed acceptable stability), rather than on the fine-grained rankings provided by the unstable bridge strength metric. Future studies with even larger sample sizes could help achieve more stable estimates of all network indices.

## Conclusion

Grounded in and providing robust empirical support for Conservation of Resources Theory, this study integrates a macro-level dose-response model with a micro-level network approach to elucidate the mechanism of the compassion fatigue-moral distress relationship. The observed nonlinear pattern and the influential bridge symptoms together paint a clear picture of a resource loss spiral in action. Our findings confirm that compassion fatigue, as a state of profound resource depletion, directly fuels moral distress. More importantly, we identify the precise levers (CF25, CF26, CF28, CF29) within this spiral that, when targeted, can most effectively disrupt its progression. Therefore, the strategic imperative informed by COR Theory is clear: interventions must be precisely designed to replenish the specific resources depleted by these core symptoms—be it through organizational reforms that restore autonomy and support (countering CF26) or individual-level therapies that process trauma and build regulatory capacity (countering CF25/28/29). Combining such targeted efforts is the most promising strategy to halt the loss spiral, conserve nurse resources, alleviate moral distress, and thereby safeguard both clinician well-being and the quality of critical care.

## Supplementary Information

Below is the link to the electronic supplementary material.

Supplementary Material 1

## Data Availability

Research data will be shared with reasonable requests with corresponding author.

## Declarations

### Patient & public involvement

No patient and public involvement during the design, conduct, reporting, interpretation, or dissemination stage of the study.
