# Supplementary material for: From depletion to distress: identifying the nonlinear relationship and core intervention target through computational simulation modelling between compassion fatigue and moral distress among ICU nurses: a cross-sectional study
Source: BMC Nurs. 2025 Dec 4;25:13. doi: 10.1186/s12912-025-04196-1 (PMC12781292; doi:10.1186/s12912-025-04196-1)
Supplement: Supplementary file 1 — Supplementary Material 1 [file 12912_2025_4196_MOESM1_ESM.docx]

Supplementary file implementation framework

This study employed a hybrid sampling strategy combining multi-center convenience sampling and snowball sampling to recruit participants. The study covered 17 geographically diverse provinces in China. In each participating province, we prioritized representative medical centers (typically tertiary Grade A hospitals) and selected their intensive care units (ICUs) as primary research sites. With the approval of each institution’s administrative department, a designated liaison (usually the head nurse or a research coordinator) was appointed to assist at each center. Liaisons performed initial screening of eligible nurses based on predefined inclusion criteria (see Section 2.3).

For primary research centers expected to recruit more than 40 participants, a trained data collector (typically a nurse with ICU experience from the respective hospital) was assigned to coordinate locally and ensure data collection quality. To address potential coverage limitations of convenience sampling, eligible participants were encouraged to recommend other qualified ICU colleagues through snowball sampling, provided the recommenders also met the inclusion criteria. Data collection was conducted from July 2023 to December 2023. All participants completed the survey via a standardized online questionnaire platform (Wenjuanxing).

All data collectors underwent standardized online training organized by the research team. The training covered the study objectives, interpretation of inclusion criteria, execution of the informed consent process, guidance on questionnaire completion, and responses to frequently asked questions to ensure standardization and consistency in the data collection process. The online questionnaire system incorporated a mandatory informed consent step. Before accessing the questionnaire, participants were required to read a detailed electronic informed consent form, which clearly outlined the study’s purpose, measures to ensure anonymity and confidentiality (emphasizing that data would only be used for aggregate analysis without identifiable personal information), the principle of voluntary participation, and the right to withdraw at any time without reason. Participants had to actively select the option “I understand the above information and voluntarily agree to participate in this study” to proceed to the questionnaire. Individuals who did not provide consent were unable to continue.

The study protocol, including sampling methods, recruitment procedures, electronic informed consent, and data security and confidentiality measures, was submitted to and approved by the ethics review committee of [Huai’an First People’s Hospital Affiliated to Nanjing Medical University] (Approval No.: KY-2023-087-01). The entire study process strictly adhered to the principles of the Declaration of Helsinki and relevant Chinese ethical regulations for human research.
